# Creating the Chinese version of the transgender attitudes and beliefs scale

**DOI:** 10.1186/s40359-024-01655-3

**Published:** 2024-03-21

**Authors:** Zhanqiang Wang, Yang Liu, Hanwen Dong, Yueqian Zhang, Kebing Yang, Qingyan Yang, Xiaolan Di, Yajuan Niu

**Affiliations:** 1 Chengde Medical University, Chengde, China; 2 Beijing Huilongguan Hospital, Huilongguan Town, Changping District, Beijing, China

**Keywords:** Transgender attitudes and beliefs scale, Reliability, Validity, Chinese university students

## Abstract

**Background:**

Trans persons’ physical and mental health is easily affected by the attitude of those around them. However, China currently lacks a valid psychometric instrument to investigate people’s attitudes toward trans persons. Therefore, this study modifies the English version of the Transgender Attitudes and Beliefs Scale (TABS) to suit the Chinese context. It subsequently examines the reliability and validity of the Chinese version of the TABS.

**Methods:**

This study recruited 1164 university students, aged 18–25 years, from 7 regions of China. SPSS26.0 and AMOS24.0 were used for data statistical analysis. Critical ratio method and correlation coefficient method were used for item analysis. Exploratory factor analysis and confirmatory factor analysis were used to test the structural validity of the Chinese version of Transgender Beliefs and Attitudes Scale, and the internal consistency reliability of the scale was tested.

**Results:**

The TABS-C contains 26 items with 3 factors. The Cronbach’s alpha was 0.957 for the total scale and 0.945, 0.888, and 0.885 for the 3 factors. The half-point reliability of the scale was 0.936, and the retest reliability was 0.877. The Pearson correlation coefficients for the 3 factors and the total scale score ranged from 0.768 to 0.946.

**Conclusion:**

The TABS-C has reliable psychometric properties and is suitable for usage among college students in the Chinese context.

## Introduction

Transgender is an umbrella term for people whose gender identity and gender expression do not match the sex assigned at birth, and who have persistent experiences of heterosexual gender identity without any pathological or sex chromosome abnormalities [1]. Including transsexuals who use or want to use hormones and/or surgery to change their gender and live full-time in their accepted gender and transgenders who often change their gender with minimal medical intervention as well as genderqueer people who sometimes switch back and forth between genders and cross-dressers who temporarily change their gender primarily through gender symbols in their appearance, they can all be collectively referred to as trans persons [2]. The trans persons may account for approximately 0.1-1.1% of the total global population [3], but the current proportion of this community in China remains unknown, According to relevant statistics, in the Asia-Pacific region, about 0.3% of adults are trans persons [4], and according to this proportion, there are about 4 million trans persons living in China, and this number is still increasing.

The experiences of trans persons around the world are varied, but one thing in common is that transsexuality is recognized as a pathology. Since gender transition has been listed as a mental disorder in the DSM and ICD since 1975, trans persons have not only faced institutional and social discrimination and violence against trans persons, but also been forced to undergo psychiatric diagnosis and evaluation [5]. In the late 20th and early 21st centuries, there have been repeated protests against the diagnosis of transsexuality as a mental disorder, arguing that defining gender diversity as a disease or other abnormality is unfounded, discriminatory, and has no demonstrable clinical utility [6]. On the basis of the right to health and the right to non-discrimination, a number of movements have emerged in support of the right to de-pathologization, the most radical of which is the Stop Trans Pathologization campaign(STP), whose main objective is to remove the gender transition process as a diagnosis of mental disorders. Transforming transgender healthcare services into an informed consent approach model that does not require legal gender recognition as required by medical treatment and has access to state-funded transgender healthcare [7]. In addition to STP, GATE [8], ILGA [9] and other campaigns have also successfully influenced the policies of countries and organizations such as the World Health Organization or the United Nations. For example, in 2013, the American Psychiatric Association published the new version of DSM-V after referring to the framework of sociology, and used “gender dysphoria” instead of “gender identity disorder” in the diagnosis of transgender [10]. Believing that removing the word “disorder” would help reduce stigma in the trans persons [11], In the International Classification of Diseases 11th Edition (ICD-11), which was subsequently published by the World Health Organization in 2018, the diagnosis of “transsexualism” was removed and all transgender-related entries were removed from the classification of “mental and behavioral disorders”, It was replaced by “gender incongruence” and moved transgender-related issues to the section “Sexual and Reproductive Health“ [12]. In addition, an increasing number of countries have revised their legislation to accommodate the non-binary classification of gender and gender [13].

Although the biopsychosocial model has improved the societal attitudes toward the trans persons to some extent [14], the change has been very gradual, and invisible barriers between the transgender and cisgender groups still exist, making the real social acceptance of trans persons difficult [15]. In many countries and regions worldwide, trans persons continue to face negative attitudes, such as implicit or explicit prejudice and discrimination [16–18], which not only hampers their self-worth, but also affects their psychological health, leading to depression, anxiety, self-harm, and suicidal thoughts or behaviors [19–22]. This seriously impairs their quality of life and survival environment, such as education, work, housing, marriage, and interpersonal communication [23].

To improve the living environment of the trans persons and eliminate prejudice, the first requirement is to learn extensively about the public attitudes toward trans persons and obtain practical research data. Doing so will not only help researchers to accurately understand public perceptions and attitudes but also help design different types of intervention programs for different groups with different attitudes. This will also be useful in providing governments with practical and effective recommendations to develop protective measures for trans persons.

A review of domestic and international research on attitudes toward trans persons in the past decade shows that researchers (especially in Europe and the United States) have developed a variety of tools to assess attitudes toward trans persons [24–29]. Current research on attitudes toward trans persons in mainland China is still in its nascent stage, where self-administered questionnaires and small-scale surveys are relied upon; there are significant limitations in terms of the reliability of research instruments and the generalizability of findings [30–31]. To make up for the shortcomings of past studies, this study chose to translate and validate the Kanamori et al. version of the Transgender Attitudes and Beliefs Scale (TABS) [27]. The TABS was translated into other languages since its English version was introduced [32]. It has been used in different regions and multiple groups [33–35], and each version has proven to be reliable. Additionally, the items in the scale contain three different factors, namely interpersonal comfort, sex/gender beliefs, and human value, that reflect the complexity of human attitudes. Therefore, this scale can not only reflect the complexity of human attitudes, but also consider people’s possible cognitive evaluation and emotional response to trans persons [27].

In view of the persistent prejudice and discrimination experienced by the trans persons and the lack of appropriate and reliable research tools in mainland China, this study revised the TABS scale using Chinese university students as a sample group to provide a scientific measurement tool for subsequent related research.

## Methods

### Participants

The Ethics Committee of Beijing Huilongguan Hospital (2023-5-Section) approved this study, and all the study procedures complied with ethical standards. The questionnaires were distributed through the online survey system “Questionnaire Star.” The inclusion criteria were as follows: university students, ability to understand the meaning of the items expressed, and voluntary responses. A total of 1,470 questionnaires were distributed. After obtaining the corresponding data, 1,164 valid questionnaires were collected after excluding the questionnaires with missing answers, regular responses, and the ones that reflected non-compliance with instructions. The recovery rate was 79.18%. All the participants filled in the questionnaire voluntarily without any remuneration, and they could submit the questionnaire only after answering all the questions. The participants were from seven regions in China: North China, Northeast China, South China, Northwest China, Central China, Southwest China, and South China.

### Research tools

For this study, the TABS by Kanamori et al. was selected [27]. It contains 29 items and 3 factors, namely interpersonal comfort (level of comfort in social interaction with trans persons), sex/ gender beliefs (whether gender is believed to be dichotomous), and human value (recognition of the intrinsic value of trans persons as human beings). It uses a seven-point Likert scale (1 = strongly disagree to 7 = strongly agree) to assess people’s attitudes toward trans persons. Higher scores represent more positive attitudes. The internal consistency of the scale was high, with Cronbach’s α for each aforementioned dimension being 0.97, 0.95, and 0.94, respectively. After obtaining the permission of the original author, the original scale was revised in Chinese. Initially, according to the Chinese cultural background and expression mode, the original scale was translated and re-translated several times by two master students in psychology and two chief psychiatrists to form Scale A without changing the meaning of the items. After that, two chief physicians in the field of sexual psychology, two associate chief physicians in the field of psychiatry and one professor in the field of psychometrics checked and modified the semantic expression and comprehensibility indicators of scale A to improve the accuracy and fluency of the scale in terms of vocabulary, grammar and other expressions, and then formed Scale B. Then, 15 individuals from different backgrounds were selected by convenient sampling method for pre-investigation. Their opinions on each item in Scale B were collected, and the order and expression of the items were revised again after summarizing the suggestions of all parties, and the Chinese version of the transgender attitude and Belief scale TABS-C was finally formed, with the same scoring method as the original scale.

### Statistical methods

SPSS26.0 and AMOS24.0 were used for statistical analysis. The total samples were used for project analysis and internal consistency reliability, sample 1 was used for exploratory factor analysis and sample 2 was used for confirmatory factor analysis. In order to test the retest reliability of TABS, 60 college students were randomly selected from 1164 Chinese college students. The measurements were taken twice every two weeks. Independent sample t test was used to analyze gender differences. Test standard α = 0.05.

## Results

### Basic information of the study participants

This study included 1164 college students from 7 regions of China, aged 18–25 years (420 males and 744 females). There were 1078 Han Chinese and 91 ethnic minorities; 389 were from cities, 287 were from counties, and 488 were from villages; 34 were religious believers and 1130 were non-believers.

### Project analysis

#### Critical ratio method

The critical ratio method was used to test the differentiation of items, and the total scores of the scale were ranked in ascending order, with the low grouping being the first 27% (314 cases) and the high grouping being the last 27% (314 cases), with a decision value of 8.240-38.272 (*P* < 0.001). This indicated good differentiation between items, and all current items were retained for the time being, as detailed in Table 1.

**Table 1:** Score comparison between high-score and low-score groups(N1 = 314, N2 = 314)

Items	Low-score group N1(M ± SD)	High-score group N2(M ± SD)	t	P
1	3.74 ± 1.688	5.33 ± 2.333	-9.798	< 0.001
2	4.81 ± 1.756	6.89 ± 0.642	-19.739	< 0.001
3	3.27 ± 1.786	6.05 ± 1.401	-21.654	< 0.001
4	3.30 ± 1.829	6.21 ± 1.240	-23.337	< 0.001
5	2.42 ± 1.494	5.79 ± 1.330	-29.851	< 0.001
6	3.39 ± 1.770	6.52 ± 1.055	-26.890	< 0.001
7	3.37 ± 1.651	6.35 ± 1.089	-26.648	< 0.001
8	3.32 ± 1.409	6.69 ± 0.672	-38.272	< 0.001
9	3.54 ± 1.480	6.80 ± 0.448	-37.320	< 0.001
10	3.39 ± 1.415	6.39 ± 1.037	-30.262	< 0.001
11	2.71 ± 1.487	5.65 ± 1.467	-24.880	< 0.001
12	4.95 ± 1.523	6.91 ± 0.433	-22.026	< 0.001
13	4.10 ± 1.432	5.75 ± 1.578	-13.719	< 0.001
14	3.36 ± 1.595	6.48 ± 1.040	-29.079	< 0.001
15	2.95 ± 1.657	6.31 ± 1.144	-29.601	< 0.001
16	2.71 ± 1.526	5.69 ± 1.593	-23.992	< 0.001
17	3.30 ± 1.771	6.45 ± 0.914	-27.941	< 0.001
18	3.76 ± 1.626	5.17 ± 2.570	-8.240	< 0.001
19	3.10 ± 1.518	6.42 ± 1.167	-30.768	< 0.001
20	3.49 ± 1.424	6.55 ± 0.911	-32.087	< 0.001
21	3.05 ± 1.605	5.60 ± 1.657	-19.521	< 0.001
22	5.03 ± 1.655	6.90 ± 0.352	-19.607	< 0.001
23	3.39 ± 1.607	6.20 ± 1.169	-25.075	< 0.001
24	3.35 ± 1.492	6.49 ± 0.996	-31.075	< 0.001
25	3.37 ± 1.346	6.59 ± 0.754	-37.021	< 0.001
26	5.02 ± 1.635	6.96 ± 0.192	-20.875	< 0.001
27	5.21 ± 1.640	6.92 ± 0.357	-18.020	< 0.001
28	3.19 ± 1.366	6.50 ± 0.967	-35.074	< 0.001
29	3.38 ± 1.588	6.42 ± 1.040	-28.445	< 0.001

#### Item-total score correlations for the TABS-C

The correlation between the scores of each entry and the total score was calculated, and if the correlation between the entry and the total score reached a significance level and *r* > 0.4, it meant that the correlation coefficient was acceptable; conversely, the item was considered for deletion [36]. The results showed that the r-values of items 1 and 18 were less than 0.4, 0.323, and 0.282, respectively, which were weakly correlated. Therefore, we consider deleting the above two items. The correlation coefficient values between the scores of other items and the total score were greater than 0.4 and *p* < 0.01, indicating that they could better respond to the content of the questionnaire. Table 2 provides more information.

**Table 2:** Item-total score correlations for the TABS-C (*N* = 1164)

Items	M ± SD	r	P
1	4.53 ± 2.017	0.323	< 0.01
2	6.09 ± 1.485	0.619	< 0.01
3	4.66 ± 1.947	0.597	< 0.01
4	4.91 ± 1.839	0.658	< 0.01
5	4.06 ± 1.883	0.714	< 0.01
6	5.09 ± 1.838	0.690	< 0.01
7	4.83 ± 1.783	0.671	< 0.01
8	5.16 ± 1.706	0.815	< 0.01
9	5.33 ± 1.616	0.829	< 0.01
10	4.86 ± 1.683	0.721	< 0.01
11	4.15 ± 1.847	0.659	< 0.01
12	6.08 ± 1.265	0.667	< 0.01
13	4.91 ± 1.602	0.413	< 0.01
14	4.97 ± 1.833	0.703	< 0.01
15	4.72 ± 1.929	0.711	< 0.01
16	4.13 ± 1.870	0.649	< 0.01
17	4.97 ± 1.840	0.682	< 0.01
18	4.35 ± 2.080	0.282	< 0.01
19	4.78 ± 1.838	0.744	< 0.01
20	5.03 ± 1.659	0.762	< 0.01
21	4.30 ± 1.866	0.571	< 0.01
22	6.11 ± 1.311	0.624	< 0.01
23	4.85 ± 1.767	0.655	< 0.01
24	4.91 ± 1.701	0.767	< 0.01
25	5.02 ± 1.665	0.809	< 0.01
26	6.23 ± 1.260	0.675	< 0.01
27	6.30 ± 1.218	0.601	< 0.01
28	4.81 ± 1.721	0.792	< 0.01
29	4.90 ± 1.768	0.710	< 0.01

### Validity test

#### Structural validity

This study’s sample was randomly divided into two groups: Sample 1 and Sample 2. An exploratory factor analysis was performed on Sample 1 (*N* = 619) using SPSS to retain the factor loadings ≥ 0.40 and no double loading or no loading items [37]. The results of the first exploratory factor analysis obtained 3 common factors, but item 13 belonged to no load. Considering the low correlation of the item, item 13 was deleted. The second exploratory factor molecular analysis showed that the total number of items of the current scale was 26. The KMO index was 0.962, which was greater than 0.90, and the Bartlett’s spherical test χ2 = 10190.594, df = 325, *P* = 0.000 < 0.001, with statistically significant differences; the results suggested suitability for exploratory factor analysis [38].

The principal component method was used to extract the common factors with eigen root values greater than 1 after maximum variance rotation. Three common factors were obtained with a cumulative contribution of 59.672%. It can be seen that the slope starts to flatten gradually after the third factor, indicating that the three common factors can contain most of the information represented by the items. According to the content of each metric and the reference to the original scale structure, the TABS-C has three metrics, i.e., three factors, namely, interpersonal comfort, sexual/gender beliefs, and human values, which are consistent with the naming of the factors of the original scale [27]. Each factor load is shown in Table 3.

**Table 3:** Factor loadings of the TABS-C (*N* = 619)

Factors	Items	Factor 1	Factor 2	Factor 3
Interpersonal comfort	28	**0.819**		
	25	**0.763**		
	20	**0.749**		
	29	**0.735**		
	9	**0.720**		
	19	**0.715**		
	24	**0.707**		
	8	**0.702**		
	16	**0.630**		
	5	**0.599**		
	7	**0.598**		
	10	**0.438**		
Sex/ gender beliefs	15		**0.744**	
	4		**0.733**	
	3		**0.687**	
	11		**0.645**	
	17		**0.599**	
	14		**0.560**	
	21		**0.559**	
	23		**0.535**	
	6		**0.489**	
Human value	26			**0.845**
	27			**0.838**
	22			**0.803**
	2			**0.613**
	12			**0.600**

**Fig. 1:**
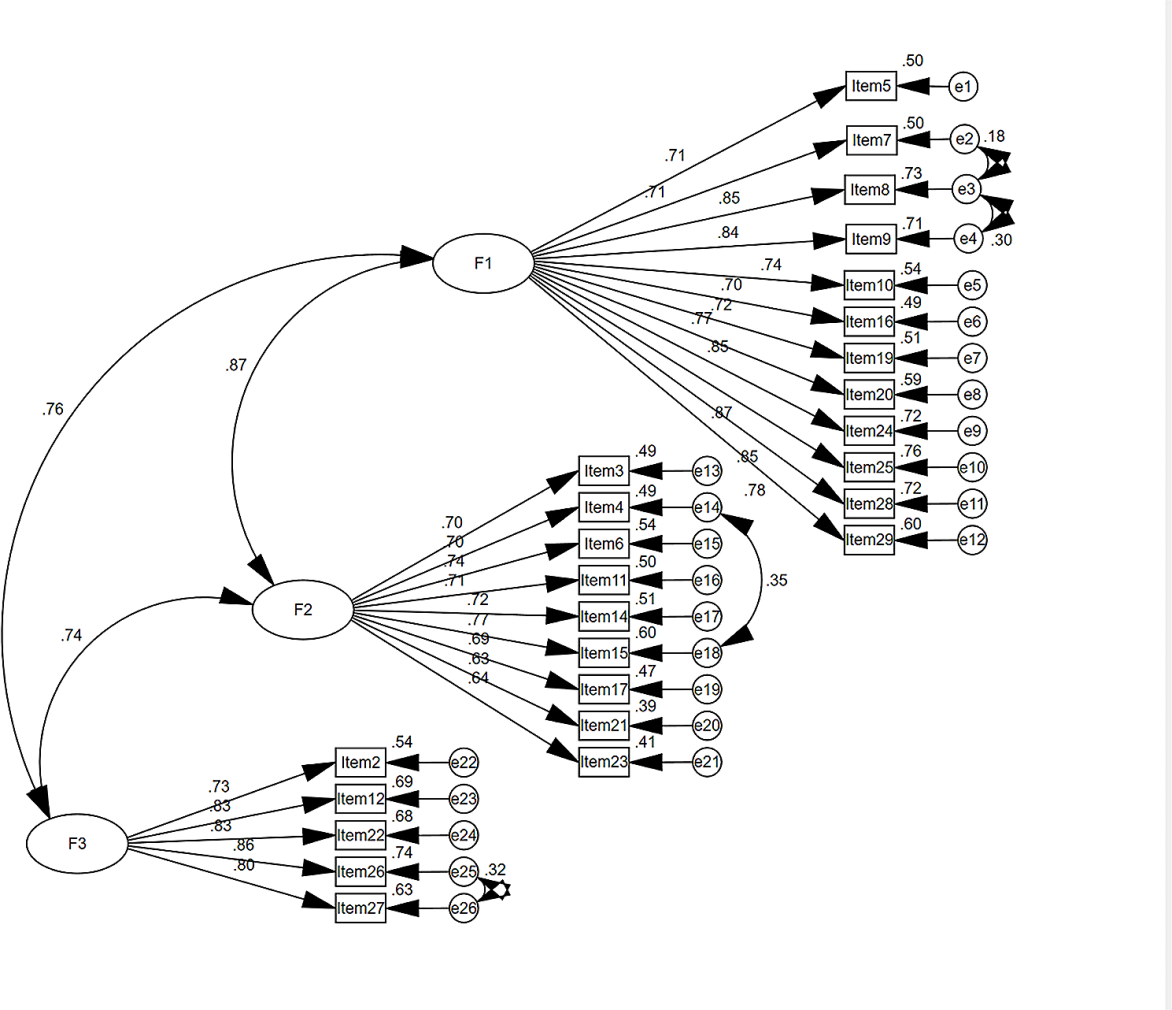
Standardized three-factor structural equation model (*n* = 545)

Validation analysis using AMOS for Sample 2 (*N* = 545), after correction of the model, showed that χ2/df, RMSEA, NFI, RFI, IFI, TLI, and CFI met the criteria [39], (χ2/df = 2.372, RMSEA = 0.050 (90% CI: 0.045–0.055), GFI = 0.904, NFI = 0.934, RFI = 0.927, IFI = 0.961, TLI = 0.956, CFI = 0.961). Table 4 shows the results, indicating that the scale has good construct validity, and the model is shown in Fig. 1.

**Table 4:** Results of the TABS-C overall model fit index (*N* = 545)

Test index	Adaptation threshold	Test result
Absolute fit index		
GFI	> 0.90	0.904
RMSEA	< 0.05	0.05
Value added fit index		
NFI	> 0.90	0.934
IFI	> 0.90	0.961
TLI	> 0.90	0.956
CFI	> 0.90	0.961
Reduced fit index		
PCFI	> 0.50	0.863
PNFI	> 0.50	0.839
NC(χ2/df)	1 < NC < 3	2.372

### Reliability analysis

The results of the reliability analysis showed that the overall Cronbach’s α was 0.957 for the TABS-C and was 0.945, 0.888, and 0.885 for each dimension, respectively. The split-half reliability of the scale is 0.936. Sixty college students were randomly selected for the test, and the interval between the 2 tests was 2 weeks. The Pearson correlation coefficients of the 3 factors and the total score of the scale ranged from 0.768 to 0.946, which were positively correlated; the correlation coefficients were statistically significant (*p* < 0.01), indicating good internal consistency of the TABS-C. Table 5 provides more information.

**Table 5:** Pearson correlation coefficient between each dimension and total score (*N* = 1164)

Factor	M ± SD	1	2	3
Interpersonal comfort	57.83 ± 16.490	-		
sex/ gender beliefs	42.61 ± 12.139	0.773^**^	-	
human value	30.82 ± 5.423	0.654^**^	0.616^**^	-
total	145.06 ± 32.652	0.946^**^	0.906^**^	0.768^**^

^**^P<0.01

#### TABS-C gender differences test

An independent samples t-test revealed significant gender-based differences, with women scoring higher across the three factors and in terms of the total score compared to men (t=-15.466, -21.060, -13.439, -19.209, *p* < 0.001). Table 6 provides more information.

**Table 6:** Gender differences in the TABS-C (*N* = 1164)

	M ± SD		t	P
	male(*N* = 420)	female(*N* = 744)		
Interpersonal comfort	48.76 ± 17.242	62.9413.613	-15.466	< 0.001
sex/ gender beliefs	34.13 ± 11.997	47.419.261	-21.060	< 0.001
human value	28.17 ± 6.765	32.313.746	-13.439	< 0.001
total	111.06 ± 32.138	142.6623.533	-19.209	< 0.001

## Discussion

This study revised the TABS developed by Kanamori et al. and explored its applicability in the Chinese context. With the consent and authorization of the original scale author, Professor Kanamori, and based strictly on Brislin’s double translation model [40], it was translated, back-translated, and revised several times to ensure equivalence between the translated and the original scale. The cultural adaptation was done based on expert consultation and pre-survey. For example, the entry in the original scale—trans persons should have the same access to housing as any other person—was included because of the social problems faced by trans persons in the United States [27]. However, this was not relevant to China. Thus, adjustments were made to make the TABS-C not only equivalent to the original scale but also more consistent with Chinese expressions and habits.

Since the scale was translated with some changes in content and form, reliability and validity tests were needed before application to see if the translation met the relevant requirements. Otherwise, it could not be applied to the local cultural context.

The findings of this study indicate that the TABS-C has good reliability and validity for use in Chinese contexts based on an optimal analysis of the scale with items 1 (“I would feel comfortable if my next-door neighbor was transgender”), 13 (“A child born with ambiguous sex-parts should be assigned to be either male or female”), 18 (“I would feel comfortable having a transgender person into my home for a meal”) being excluded. Items 1 and 18 were deleted because the correlation coefficients were too low, and item 13 could not be categorized in either factor during the exploratory factor analysis. The final TABS-C contains 26 items, and the exploratory factor analysis in structural validity yielded 3 common factors with eigenvalues greater than 1, and the cumulative contribution of variance was 59.672%. It is generally considered that the cumulative contribution of the extracted common factors to the total variance is greater than 40% [41]. The 3 common factors are F1: interpersonal comfort, F2: sex/gender beliefs, and F3: human value. F1 includes items 5, 7, 8, 9, 10, 16, 19, 20, 24, 25, 28, and 29; F2 includes items 3, 4, 6, 11, 14, 15, 17, 21, and 23; and F3 includes items 2, 12, 22, 26, and 27. The results of factor analysis showed that χ2/df, RMSEA, NFI, RFI, IFI, TLI, and CFI met the criteria, indicating that the structural equation model of the TABS-C is good and has a more stable internal structure.

Based on previous studies, Cronbach’s α < 0.6 indicates low internal consistency, 0.6–0.8 indicates fair internal consistency, and Cronbach’s α > 0.8 indicates an ideal situation [42]. The overall Cronbach’s α for the TABS-C was 0.957 and 0.945, 0.888, and 0.885 for each dimension, respectively. The split-half reliability of the scale is 0.936, and the retest reliability was 0.877. The Pearson correlation coefficients for the 3 factors and the total scale score ranged from 0.768 to 0.946, indicating high consistency and stability within the scale.

Surprisingly, the results of the current study differed from the results of the other previously validated versions. The original version of these contained 3 factors and 29 items, and the original scale was validated in the context of American Christians as well as 2 Spanish-speaking contexts, with results consistent with the original 29-item version. Although our results also support 3 factors, we speculate that the total number of items was censored to 26 due to cultural, geographical, and sample differences. This study’s sample was college students aged 18–25 years who were from different regions and majored in different subjects in China. The sample of Kanamori et al. included 207 adults with religious affiliation in the United States, aged 21–75 years [33]. The sample of Miguel et al. included 829 psychology students from 3 public universities in Spain, wherein 79% were female [32]. Kanamori et al. again selected 605 participants for their study, 38% of whom were from the United States and 62% from Spain, with an average age of 35.84 years [43]. This was far lesser than our sample size, and differences in sample representation may have caused differences in the understanding of the items. Additionally, cultural factors also played an important role, such as the elimination of “I would not feel uncomfortable if my neighbor was trans person,” probably because in China, even if they live in a flat, the neighbors may not know or interact with each other. Hence, no well-defined attitude toward a transgender neighbor develops, making this item less relevant to the overall score. The deletion of “I would not feel uncomfortable if a trans person were to come to my house for dinner” may be because in China, inviting other people to one’s family gatherings is infrequent, resulting in a lower correlation between this item and the total score. Therefore, in addition to differences in the sample, cultural context is also a key aspect to consider while translating.

Group validity refers to a measure of the ability to capture differences between groups as predicted by theoretical or empirical data [44]. Given that previous studies have consistently found that men tend to have more negative attitudes toward trans persons than women [43, 45–47], the same study that found significant differences in gender attitudes toward trans persons (total and all dimension scores reflecting higher tendency among women toward inclusion) provided evidence for group validity. As with the previous results, part of the reason for this gender difference made researchers believe that the strong identification of men with traditional gender roles compared to women and the emergence of the trans persons threatens their inherent gender binary perceptions. Therefore, to improve men’s attitudes toward trans persons, relevant intervention studies could be conducted to determine what works.

## Conclusion

The Trans Attitude and Belief Scale is a reliable and effective tool for evaluating trans attitudes in the Chinese context. It can assess college students’ attitudes toward trans persons. In addition to evaluating attitudes, the scale also provides an important multidimensional measurement factor, which is helpful for studying individuals’ views on gender and human values. These findings can be applied to the Chinese college student population, but not to other populations living in China, so the uniformity of the sample structure requires that our future studies should encourage testing of the psychometric properties of the scale in different populations in China. According to statistics, women score higher in the scale than men. In the future, we can focus on the relationship between college students’ attitudes toward transgender people and demographic factors or individual factors.

## Data Availability

The original data supporting the conclusions of this study and the Chinese version of the scale will be provided by the authors without reservation to appropriate researchers for verification. For further information, please contact author Zhanqiang Wang at 15512483861@qq.com.

## Declarations

All methods were carried out in accordance with relevant guidelines and regulations.
